# Characterization and Analysis of 2-(2-Phenylethyl)chromone Derivatives and Sesquiterpenoids from Agarwood of Four “Qi-Nan” Clones (*Aquilaria sinensis*) with Different Induction Times

**DOI:** 10.3390/molecules30020352

**Published:** 2025-01-16

**Authors:** Ming Li, Zhou Hong, Shengjie Wang, Daping Xu, Zinong Yang, Zhihui Li, Houzhen Hu, Suxin Li

**Affiliations:** 1 The Key Laboratory of National Forestry and Grassland Administration for Tropical Forestry Research, Research Institute of Tropical Forestry, Chinese Academy of Forestry, Guangzhou 510520, China; liming@caf.ac.cn (M.L.); hzhou1981@caf.ac.cn (Z.H.); xdp@caf.ac.cn (D.X.); yzn18702002724@hotmail.com (Z.Y.); li357712@outlook.com (Z.L.); hhz494@njfu.edu.cn (H.H.); 2 College of Horticulture and Landscape Architecture, Zhongkai University of Agriculture and Engineering, Guangzhou 510225, China

**Keywords:** “Qi-Nan”, 2-(2-phenylethyl)chromones, SESs, agarwood quality, GC-QTOF-MS

## Abstract

In recent years, some new “Qi-Nan” clones of *Aquilaria sinensis* with the characteristics of easy induction and high-quality agarwood have been obtained, through the cultivation and propagation of grafted seedlings. These clones are used for the intensive production of high-quality agarwood. The speed of resin formation and yield are crucial for the development of the agarwood industry. The differences in yield and chemical composition among different Qi-Nan clones and induction times are worth investigating. While the chemical composition differences between Qi-Nan and ordinary *A. sinensis* have been extensively studied, the effects of induction time coupled with different Qi-Nan clones on the chemical composition of Qi-Nan agarwood remain insufficiently explored. This study compared the changes in the chemical composition of four “Qi-Nan” clones of *A. sinensis* after 6, 12, and 24 months of induction through GC-QTOF-MS, the chemical composition and structure types of the four “Qi-Nan” clones were mainly 2-(2-phenylethyl)chromone derivatives (PECs) and Sesquiterpenoids (SESs), with the prolongation of induction time, the content of SESs increased, while the content of PECs decreased. Both the differences among clones and the induction time of “Qi-Nan” agarwood influence its chemical composition, which in turn affects the quality of the agarwood. Among these factors, induction time has a greater impact on the production of PECs in agarwood. The prolongation of induction significantly enhanced the yield of “Qi-Nan” agarwood and exhibited an inducing effect on the production of 2-(2-phenylethyl) chromone and 2-(2-4 phenylethyl)chromone. Compared with the agarwood obtained after 6 and 12 months of induction, the agarwood of “Qi-Nan” after 24 months of induction exhibited superior quality. The induction time for high-quality agarwood from the XGY clone was shorter (12 months) compared to the RH, YYZ, and AS clones (24 months). The study underscores that optimizing induction time and selecting suitable “Qi-Nan” clones can significantly enhance agarwood yield, quality, and production efficiency.

## 1. Introduction

*A. sinensis* is a tropical-subtropical evergreen tree, which is mainly distributed in South and Southeast Asia. In China, it is mainly distributed in Guangdong, Hainan, Guangxi, Fujian and other provinces [1]. Agarwood is a resinous heartwood from the Aquilaria and Gyrinops species of the Thymelaeaceae family [2], and its formation stems from the response of trees to various forms of injury, either by natural injuries (e.g., lightning strikes, animal grazing, insect attacks, or microbial invasions) or by anthropogenical (e.g., cutting, nailing, artificial holing, fires, chemical injuries, artificial fungal inoculation, etc.) [3,4,5]. The formation of agarwood involves the accumulation of secondary metabolites, mainly SESs and PECs, which confer its distinct aroma and biological activities [1,2].

Agarwood, a highly valued traditional herb and spice, has become scarce due to overexploitation of wild Aquilaria species and its naturally lengthy formation process spanning decades or centuries [6]. Artificial induction is now the main method of production, with most commercial agarwood derived from cultivated trees, where artificial holing is the most common technique [6]. Among the commercially exploited species, *A. sinensis*, *Aquilaria malaccensis* Lam., *Aquilaria crassna* Pierre ex Lecomte and more recently *Aquilaria filaria* (Oken) Merr. are prominent, with “Qi-Nan” agarwood (e.g., Kanankoh or Kyara) regarded as the highest quality due to its rarity and superior characteristics, commanding prices significantly higher than ordinary agarwood [2,7]. To meet demand, high-quality “Qi-Nan” clones (e.g., YYZ, XGY, AS, RH) have been propagated via grafting, producing resin-rich agarwood in shorter times under artificial induction. Factors such as germplasm type and induction duration critically affect the yield and quality of agarwood [8].

The two main classes of characteristic components in agarwood are SESs and PECs [1,9], and as of 2019, a total of 182 SESs and 240 PECs have been isolated and identified from agarwood [2,10,11,12]. They are widely regarded as the primary markers for evaluating its quality. PECs are highly characteristic chromones that are responsible for the unique fragrance and pharmacological properties of agarwood, including anti-inflammatory, neuroprotective, and antioxidant activities [13,14,15]. On the other hand, SESs, especially sesquiterpene hydrocarbons and oxygenated derivatives, contribute to the aroma and biological activities, including antimicrobial and anticancer properties [16,17,18]. The relative proportions of PECs and SESs are essential determinants of the chemical profile, fragrance, and biological value of agarwood.

While numerous studies have focused on the mechanisms of agarwood formation in *A. sinensis* and comparisons between ordinary and “Qi-Nan” agarwood, there has been little research investigating the differences among “Qi-Nan” clones [19]. For instance, Chen et al. analyzed the chemical composition of agarwood in *A. sinensis* and highlighted significant differences in SESs and PECs between natural and artificially induced agarwood, but the study was limited to ordinary agarwood [3]. Similarly, Yang et al. identified the unique chemical profiles of “Qi-Nan” agarwood, such as higher proportions of PECs, demonstrating its superior quality, but no distinction was made between different “Qi-Nan” clones [7]. In addition, Li et al. focused on the biosynthetic pathways of agarwood compounds and compared ordinary and “Qi-Nan” agarwood, emphasizing the need for further investigation into the genetic and metabolic differences among “Qi-Nan” clones [10]. Given the increasing importance of “Qi-Nan” agarwood in the market, it is necessary to evaluate the variations in agarwood formation among different “Qi-Nan” clones.

Moreover, induction time is a critical factor influencing the chemical composition and quality of agarwood [20]. Studies by Ma et al. and Chen et al. revealed that prolonged induction time significantly affects the accumulation of SESs and PECs, with substantial changes observed at 6, 12, and 24 months in ordinary agarwood [8,21]. Additionally, Tian et al. demonstrated that induction time impacts not only the yield but also the ratios of key compounds, such as SESs and PECs, in *A. sinensis*. However, their work focused on natural and artificially induced agarwood without examining “Qi-Nan” clones [1]. Despite these advances, previous studies have not considered the combined effects of induction time and “Qi-Nan” clones. The coupling of these two factors remains unexplored and warrants systematic investigation to better understand their interplay in determining agarwood quality.

In this study, the chemical compositions of four “Qi-Nan” clones with different induction times were detected and analyzed by gas chromatography-quadrupole time-of-flight mass spectrometry (GC-QTOF-MS), and the relative contents of SESs and PECs of “Qi-Nan” agarwood were compared in detail after 6, 12, and 24 months of induction, which revealed the accumulation patterns of SESs and PECs in the agarwood at different induction times. It is not only conducive to the identification of “Qi-Nan” agarwood with different formation time and establishment of the quality evaluation system, but also conducive to the promotion of the cultivated “Qi-Nan” agarwood lines in the future and the further improvement of the technology level of the deep processing and utilization of agarwood.

## 2. Results

### 2.1. Ethanol Extract Content Analysis

**Figure 1:**
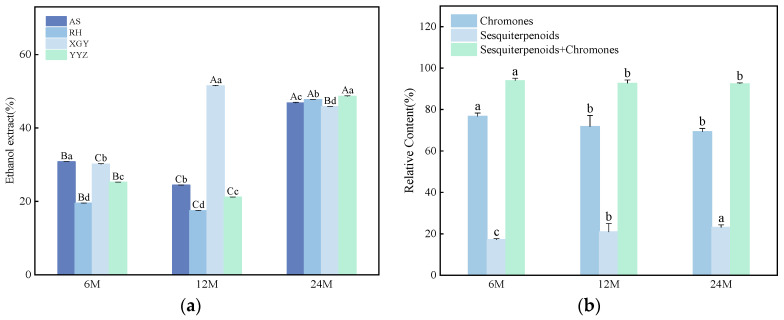

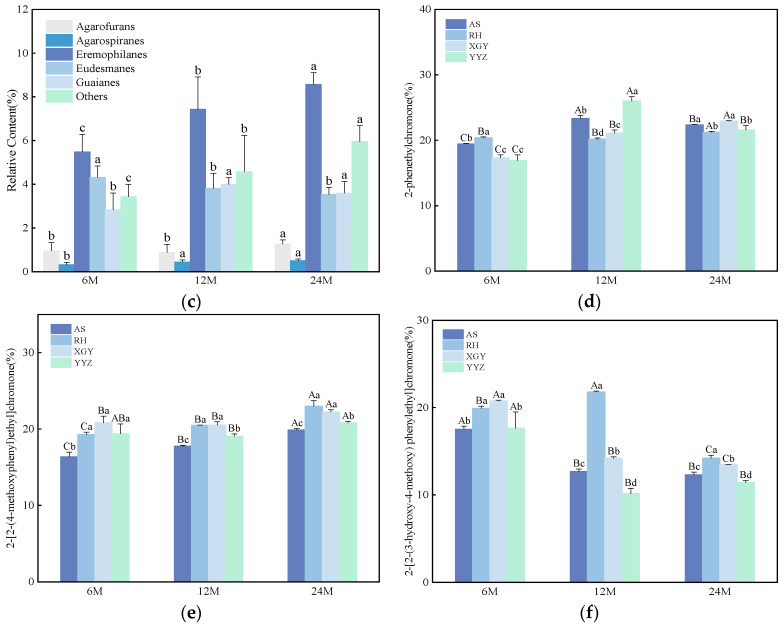
(**a**) Ethanol extract content; (**b**) The relative contents of PECs and SESs; (**c**) The relative contents of six major types of sesquiterpenoids; (**d**) 2-(2-phenylethyl)chromone relative content; (**e**) 2-[2-(4-methoxyphenyl)ethyl]chromone relative content; (**f**) 2-[2-(3-hydroxy-4-methoxy)phenylethyl]chromone relative content. Note: Data are presented in the mean ± SE. Different capital letters indicate that the significant differences among induction times at the *p* < 0.05 level. Different small letters indicate that the significant differences among four “Qi-Nan” clones at the *p* < 0.05 level. AS, RH, XGY, YYZ are “Qi-Nan” clones of *A. sinensis*. 6 M: induced for 6 months; 12 M: induced for 12 months; 24 M: induced for 24 months.

The resin content of agarwood is a key quantitative criterion for grading the quality of agarwood [22]. In the present experiment, four “Qi-Nan” clones were extracted with ethanol using cold immersion sonication method. The results showed that the average ethanol extract content of the four “Qi-Nan” clones was 26.41 ± 5.24%, 28.64 ± 15.50%, and 47.23 ± 1.18% after 6, 12, and 24 months of induction, respectively. Notably, the ethanol extract content of the XGY clone (51.49%) peaked at 12 months of induction, which was significantly higher than that of the other clonal lines and other induction times. Meanwhile, the ethanol extract content of the YYZ (48.59%), AS (46.82%), and RH (47.68%) clones demonstrated superior quality after 24 months of induction, which was noticeably higher compared to their respective results at 6 and 12 months of induction (Figure 1a). To summarize, the prolongation of induction time significantly enhanced the ethanol extract content of “Qi-Nan” agarwood. However, the optimal induction time varied among clones. The induction time for high-quality agarwood from the XGY clone was shorter (12 months) compared to the RH, YYZ, and AS clones. Therefore, we recommend harvesting high-quality agarwood from the XGY clone after 12 months of induction, while for the YYZ, AS, and RH clones, extending the induction time is advisable to achieve superior quality.

### 2.2. GC-MS Analysis of SESs and PECs from Agarwood of Four “Qi-Nan” Clones

In order to elucidate the changes in chemical composition during agarwood formation, the ethanol extracts of four “Qi-Nan” clones at three stages of induction were analyzed by GC-QTOF-MS. A total of 63 compounds were identified across the four agarwood clones at different induction times. Among these, SESs and PECs were identified as the primary components of agarwood. In addition, trace amounts of esters, alcohols, acids, phenols, and triterpenoids were also detected. The identified fractions are listed in Table 1. The relative contents of PECs after 6, 12, and 24 months of induction were 76.78 ± 1.55%, 71.82 ± 5.30%, and 69.35 ± 1.50%, respectively, for the four “Qi-Nan” clones. Conversely, the relative contents of SESs were lower at 17.18 ± 0.57%, 20.92 ± 4.06%, and 23.14 ± 1.22%, respectively (Figure 1b). Over time, as the induction period prolonged, the relative content of SESs increased, while the relative content of PECs decreased.

**Figure 2:**
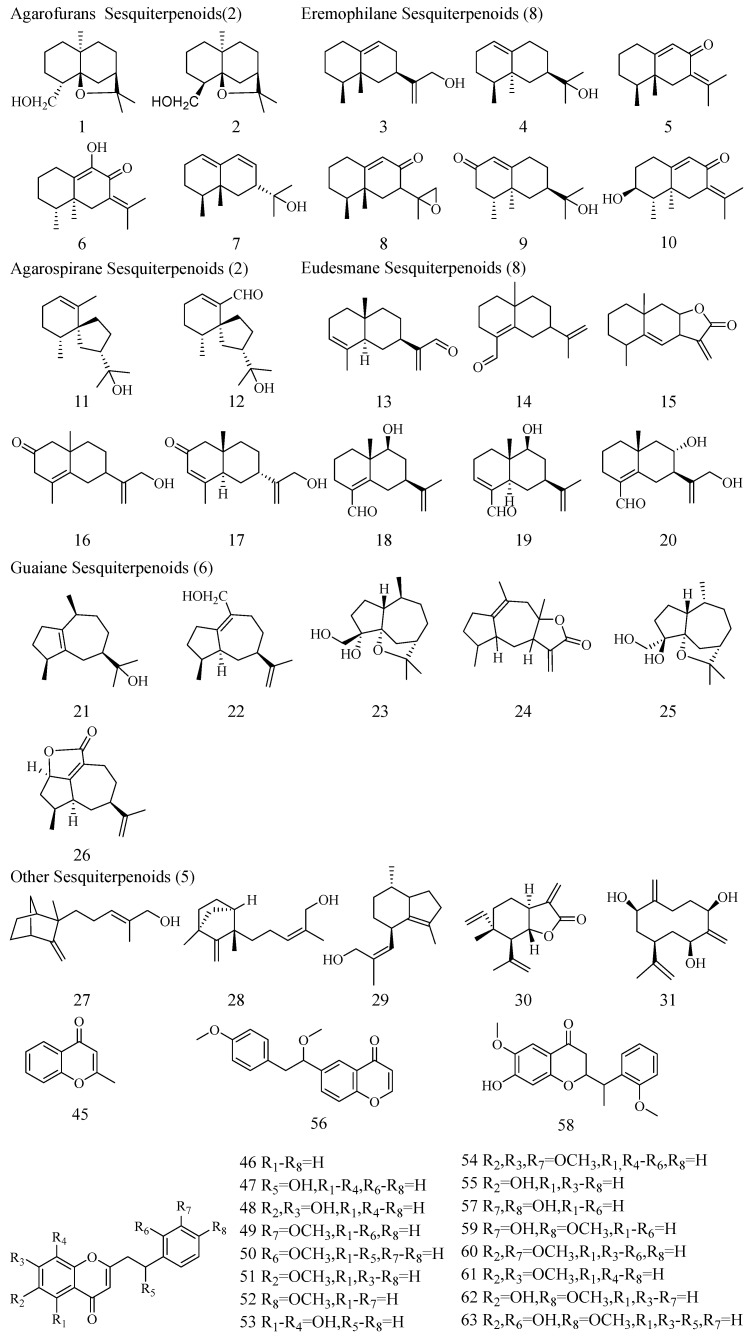
Structures of Sesquiterpenoids and Chromones identified in agarwood of four “Qi-Nan” clones. Note: The serial numbers below the structural diagrams of the chemical compositions correspond to the serial numbers of the chemical compositions in Table 1.

SESs isolated from agarwood exhibit a variety of types, including Eudesmanes, Agarospiranes, Eremophilanes, Guaianes, and Agarofurans types (Figure 2) [10]. SESs, as one of the characteristic components of agarwood, are dominated by Eremophilanes and Eudesmanes. They appear relatively early and in low relative amounts. Obviously, the contents of Agarospiranes, Eremophilanes, Guaianes, and Agarofurans SESs exhibited a general upward trend with increasing induction time, reaching their highest levels at 24 months. In contrast, Eudesmanes SESs, represented by 8,12-dihydroxy-selina-4,11-dien-14-al (20) and eudesma-3,11(13)-dien-12-al (13) decreased with increasing induction time. (Figure 1c) PECs are considered to be responsible for the quality of agarwood, which can be broadly categorized into the following four types: Flidersia type (FTPECs), 5,6,7,8-tetrahydro-2-(2-phenylethyl)chromones (THPECs), 5,6-epoxy-2-(2-phenylethyl)chromones (EPECs), and 5,6,7,8-diepoxy-2-(2-phenylethyl)chromones (DEPECs) [23], in this study, FTPECs type were widely distributed and accumulated most in “Qi-Nan” agarwood. All four “Qi-Nan” clones exhibited high levels of PECs at all three induction times, especially 2-(2-phenylethyl)chromone (46) (Figure 1d), 2-[2-(4-methoxyphenyl)ethyl]chromone (52) (Figure 1e), and 2-[2-(3-hydroxy-4-methoxy)phenylethyl]chromone (59) (Figure 1f) were the highest in abundance, which are characteristic compounds of “Qi-Nan” agarwood [23]. The relative content of the three chromones cumulatively reached 56.42 ± 3.08%, 56.78 ± 3.57%, and 56.33 ± 2.36% after 6, 12, and 24 months of induction, respectively. The increase in induction time had an inducing effect on the production of (46) and (52), while it had an inhibiting effect on the production of (59).

### 2.3. Multivariate Statistical Analysis Revealed Metabolic Differences Between Induction Times and Clones of “Qi-Nan” Agarwood

#### 2.3.1. Principal Component Analysis and K-Means Clustering Analysis

**Figure 3:**
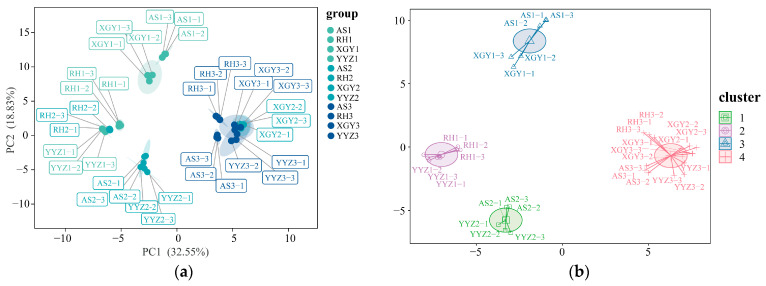
(**a**) PCA model of the distribution of chemical constituents in the agarwood of four “Qi-Nan” clones from *A. sinensis*; (**b**) KCA model of the distribution of chemical constituents in the agarwood of four “Qi-Nan” clones from *A. sinensis*. YYZ1, YYZ2, YYZ3: YYZ after artificial inducing for 6, 12, 24 months, YYZ1-1, YYZ1-2, YYZ1-3 are three YYZ replicates after artificial inducing for 6 months, respectively, with similar naming rules applied to AS, RH, and XGY clones.

The chemical constituents of “Qi-Nan” agarwood at different induction times were downgraded and grouped by Principal Component Analysis (PCA) (Figure 3a) and K-Means Clustering Analysis (KCA) (Figure 3b). The chemotypes of each cluster were categorized according to the advantages of the compounds at different induction times of “Qi-Nan” agarwood. PCA showed that the first principal component (PC1) and the second principal component (PC2) explained 51.38% of the total variance in the induction time of “Qi-Nan” agarwood. PCA and KCA analyses classified “Qi-Nan” agarwood with three different induction times into four different taxa. Taxon I included AS and XGY clones induced for 6 months; RH and YYZ clones induced for 6 months, and RH clones induced for 12 months were classified as taxon II; AS and YYZ induced for 12 months were categorized as taxon III; and the four “Qi-Nan” clones induced for 24 months and XGY clone induced for 12 months were categorized as taxon IV. The four taxa were not classified according to the “Qi-Nan” clones or the induction time. The samples induced for 6 and 12 months were distributed on the negative axis of PC1, whereas the four “Qi-Nan” clones induced for 24 months and the XGY clone induced for 12 months were mainly clustered on the positive axis of PC1, exhibiting high levels of (20), 6-methoxy-2-(2-phenylethyl)chromone (51), (52), 2-[2-(3-methoxy-4-hydroxy)phenylethyl]chromone (57), (59), these five compounds were the important compounds affecting the grouping. The PCA results showed that there was a significant difference in compound content between “Qi-Nan” agarwood induced for 24 months and those induced for 6 and 12 months. Additionally, the XGY clone induced for 12 months could reach the standard of “Qi-Nan” agarwood induced for 24 months. The results of the KCA aligned with the classifications obtained from the PCA. PCA and KCA revealed that in addition to different clones, the time of induction may also affect the metabolic characteristics of “Qi-Nan” agarwood.

#### 2.3.2. Orthogonal Partial Least-Squares Analysis

**Figure 4:**
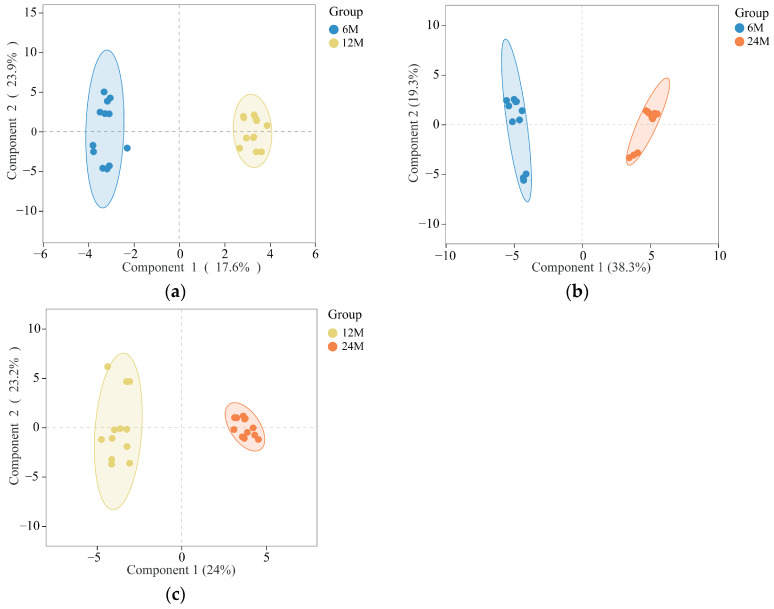
(**a**) Orthogonal partial least-squares (OPLS) model of the chemical constituent’s distribution of “Qi-Nan” agarwood induced for 6 months and 12 months; (**b**) Orthogonal partial least-squares (OPLS) model of the chemical constituents distribution of “Qi-Nan” agarwood induced for 6 months and 24 months; (**c**) Orthogonal partial least-squares (OPLS) model of the chemical constituents distribution of “Qi-Nan” agarwood induced for 12 months and 24 months.

In order to identify the differentiated compounds, the data were further processed by Orthogonal Partial Least-Squares Analysis(OPLS-DA).The 6 M vs. 12 M group [R^2^Y(cum) = 0.988; Q^2^(cum) = 0.953] (Figure 4a), the 6 M vs. 24 M group [R^2^Y(cum) = 0.987; Q^2^(cum) = 0.983] (Figure 4b), the 12 M vs. 24 M group [R^2^Y( cum) = 0.983;Q^2^(cum) = 0.924] samples are clearly separated in Figure 4c; R^2^ and Q^2^ are important parameters of the model, indicating how much the data contributes to the creation of the model and the degree of prediction. The closer R^2^ and Q^2^ are to 1, the better the model is. In order to prevent the model from overfitting, a replacement test with 200 iterations was conducted. The R^2^Y and Q^2^Y of the three Orthogonal Projections to Latent Structures Discriminant Analysis(OPLS-DA) were greater than 0.9, and the results of the 200-replacement test proved that the predictive ability of the OPLS-DA model was reliable to a certain extent, which meant that “Qi-Nan” agarwood could be clearly distinguished at different induction times. The OPLS-DA results explained the differences in compound distributions among “Qi-Nan” agarwood induced for 6, 12, and 24 months. To further explore the differential metabolites of “Qi-Nan” agarwood at different induction times, the differential metabolites were screened according to the Variable Importance Projection (VIP > 1) of each metabolite in the OPLS-DA model combined with the Fold Change (FC > 2 or FC < 0.5) and the *p*-value (*p* < 0.05), and the VIP values reflected the influence of each compound on the classification, and the variables with the VIP values of > 1 had a moderate to high influence on the Y-matrix of the interpretation had a moderately high impact [24], resulting in a total of 15 differential metabolites screened, including seven PECs, suggesting that these 2-(2-phenylethyl)chromones may be characteristic compounds that are capable of distinguishing agarwood with different induction times.

**Figure 5:**
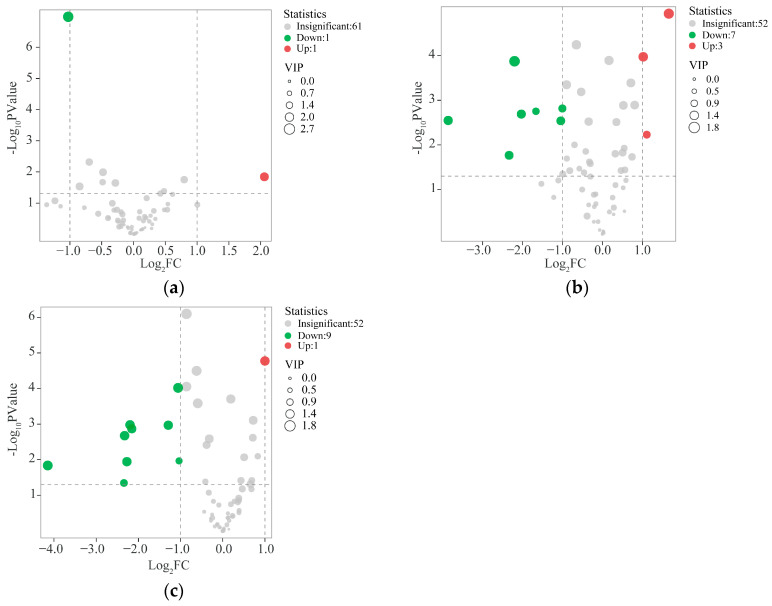
(**a**) Volcano diagram of “Qi-Nan” agarwood induced for 6 months and 12 months; (**b**) Volcano diagram of “Qi-Nan” agarwood induced for 12 months and 24 months. (**c**) Volcano diagram of “Qi-Nan” agarwood induced for 6 months and 24 months.

Further visualization of the differential metabolites, as can be seen from the volcano plot, there were two differential metabolites in the induced 6 and 12 months group, of which one metabolite was up-regulated to accumulate in the 12 months group, and one metabolite was significantly down-regulated after 12 months (Figure 5a). There were 10 differential metabolites, identified in the induced 6 and 24 months group, of which three metabolites were up-regulated to accumulate after 24 months of induction, and seven metabolites were significantly down-regulated after 24 months of induction (Figure 5b), There were 10 differential metabolites in the induced 12- and 24-month groups, of which 1 metabolite was up-regulated and accumulated in the middle of 24 months after induction, and 9 metabolites were significantly down-regulated after 24 months of induction (Figure 5c). The 15 differential metabolites were the key factors in the formation of “Qi-Nan” agarwood with different induction times. Notably, the compound 6,7-dimethoxy-2-phenethylchromone was identified as a shared differential metabolite between the 12 M vs. 24 M and 6 M vs12 M groups, (13), stearic acid (32), benzenepropanoic acid, 2-phenylethyl ester (42), 2-hydroxy-2-(2-phenylethyl)chromone (47), 6,8-dihydroxy-2-(2-phenylethyl)chromone (48), 2-[2-(3-methoxy)phenylethyl]chromone (49) were identified as common differential metabolites between the 12 M vs. 24 M and 6 M vs. 24 M groups, indicating that there was a significant difference in the content of these compounds between the induction times of 6, 12, and 24 months, and these six compounds could be used as candidate marker compounds to distinguish different “Qi-Nan” agarwood samples harvested at different formation times.

### 2.4. Factors Affecting the Formation of PECs in “Qi-Nan” Agarwood

The results of two-factor main effect analysis (Table 2) showed that the two factors of induction time and clonal line had highly significant main effects on the total content of “Qi-Nan” agarwood chromones, indicating that both induction time and clonal line had highly significant effects on the formation of PECs of “Qi-Nan” agarwood, with induction time having the largest F-value, which was the main factor, and the clonal line of “Qi-Nan” agarwood was the secondary factor; and the interaction of the two factors of induction time and clonal line also had highly significant effects on the content of “Qi-Nan” agarwood.

## 3. Discussion

### 3.1. Variation in Ethanol Extract Yields and Quality of “Qi-Nan” Agarwood Clones Induced by Artificial Holing Method

The ethanol extract content of “Qi-Nan” agarwood varied significantly among clones and induction periods. Among the four clones studied, XGY demonstrated the highest ethanol extract yield after 12 months of artificial holing induction, significantly outperforming YYZ, RH, and AS. By 24 months, the average ethanol extract content of XGY, YYZ, RH, and AS exceeded 45%, a level unattainable in ordinary *A. sinensis* germplasm under similar conditions [25]. Notably, the ethanol extract content of these clones approached that of wild “Qi-Nan” agarwood (49.85%) and was substantially higher than ordinary agarwood, consistent with previous findings [23]. XGY clone exhibited the fastest resin accumulation, reaching peak ethanol extract content within 12 months, whereas YYZ, RH, and AS required 24 months to achieve optimal quality. These results align with Li et al., who observed peak resin formation in “Qi-Nan” agarwood after two years of induction [26]. Extending the induction time notably improved resin yields in slower resin-forming clones. Artificial holing induction proved to be an effective technique for high-quality agarwood production, enabling rapid resin formation and stable alcohol-soluble leachate accumulation. After just 6 months of induction, all four clones produced high-quality agarwood, with further quality improvements observed at 24 months. Additionally, combining artificial holing with fire, fungal inoculation, or salt treatments further enhanced agarwood yield and quality [27,28]. In summary, the induced agarwood from XGY, YYZ, RH, and AS clones sharing similar characteristics with wild “Qi-Nan” agarwood in high content of ethanol extracts and other characteristics, highlighting its significant commercial potential. Therefore, optimizing harvesting strategies based on the specific resin-forming traits of each clone is critical to maximizing agarwood quality and yield.

### 3.2. The Characteristics of SESs and PECs of “Qi-Nan” Agarwood During Induction Period

The most abundant ethanol extracts of the four “Qi-Nan” clones were (46), (52), and (59), which were similar to the results of the ethanol extracts of “Qi-Nan”, which have been reported by researchers such as Li et al. [26], Chen et al. [21]. The concentrations of these three chromones were significantly higher—hundreds of times greater—than those found in ordinary agarwood. This remarkable difference helps explain the high ethanol extract content of “Qi-Nan” agarwood observed in previous studies. Numerous studies have shown that (46), (52), and (59) are the characteristic compounds of “Qi-Nan” agarwood [23]. Furthermore, many of the PECs exhibit a wealth of biological activities, which are valuable for in-depth research.

The content of PECs of agarwood can be used to judge the formation time or quality of agarwood [9]. In this study, SESs and PECs dominated the four “Qi-Nan” clones agarwood, with PECs being simpler and the highest concentration of FTPECs, which was in agreement with Yu et al. [24]. Recent studies have shown that FTPECs are precursors of THPECs and EPECs, and PECs are further modified in biosynthesis by hydroxylation, methylation, isomerization, epoxidation, halogenation, etc., and these post-modification reactions greatly increase the diversity of PECs species and structures. Due to the higher expression levels of key enzymes in the biological pathway for synthesizing FTPECs in “Qi-Nan” agarwood compared to ordinary agarwood, and the shorter metabolic pathway, “Qi-Nan” agarwood has a greater advantage in enriching the components of FTPECs [21].

The increase in total sesquiterpene content in “Qi-Nan” agarwood over time, coupled with a decline in specific Eudesmanes SESs (e.g., (20) and (13)), can be attributed to the dynamic metabolic processes and enzymatic pathways involved in agarwood resin biosynthesis. As the induction process progresses, significant changes occur in the expression of terpene synthases and other related enzymes. The transformation of primary sesquiterpenes into more oxygenated or complex derivatives is facilitated by key enzymes, including cytochrome P450s and terpene synthases (TPS) [29]. This enzymatic activity leads to the gradual depletion of Eudesmanes SESs as they are utilized in downstream biosynthetic pathways [30]. Acting as precursors, Eudesmanes SESs are metabolized into increasingly complex or oxygenated sesquiterpenes, which further enhance the chemical complexity of the resin [31].

### 3.3. Exploration of the Mechanism of “Qi-Nan” Agarwood Formation

The induction of agarwood is a complex and dynamic process. Upon injury or damage to an agarwood tree, microbial infestation frequently occurs concurrently and can persist over an extended period. Therefore, many studies have suggested that the formation of incense in agarwood trees is the result of a combination of external damage and microorganisms, especially the endophytic fungi of agarwood trees [32]. During the induction process, carbohydrates decrease while metabolites of agarwood increase [33], and these specific metabolites promote plant wound healing and anti-microbial resistance while forming agarwood [34].

Ma et al. analyzed agarwood of ordinary germplasm of *A. sinensis* induced by inoculation for 6, 12, and 18 months by GC-MS, and found that the relative content of SESs increased with the prolongation of inoculation time. By investigating the effects of biological treatments (e.g., fungal inoculation), chemical treatments, and mechanical treatments on the content of sesquiterpenes in agarwood [35], Le et al. demonstrated that the relative content of SESs increased significantly with the increasing time of agarwood formation [36]. In our study, SES accumulation began one year after induction and reached its peak by the second year, marking a critical phase for sesquiterpene biosynthesis., which was in agreement with the results of previous studies. This is likely linked to long-term metabolic changes within the wood and the tree’s defense mechanisms [30,37]. SES accumulation serves as a defense mechanism. Activated genes encode enzymes that facilitate the production of sesquiterpenes through the mevalonic acid (MVA) and methylerythritol phosphate (MEP) pathway. Key enzymes, such as farnesyl pyrophosphate synthase (FPPS) and sesquiterpene synthases, catalyze the formation of various sesquiterpene compounds [38].

Chen et al. performed GC-MS analysis of the chemical constituents of ordinary agarwood before and up to 12 months after induction, and the results showed that PECs were first detected after 2 months of induction, with most of the PECs detected only after 4 months, and the concentration of PECs progressively increased over the induction time and reached a peak at 12 months [32]. Ma et al. found that the chromone content increased continuously from 6 to 12 months during the induction process of the ordinary germplasm of *A. sinensis*, but declined when the induction time exceeded 12 months [35]. In the present study, the relative content of chromone derivatives in the “Qi-Nan” clones decreased with the increase of induction time, but it remained the component with the highest relative content, which is consistent with the results of Li et al. [26].This could be attributed to the fact that the xylem was infested by the fungus during 6–12 months of induction, which led to the activation of plant defense responses, alteration of primary and secondary metabolism, degradation of starch analogs, and production of synthesized PECs in the dead cells to resist the fungal damage, resulting in an increased content of PECs [35]. However, during the ongoing defense process, certain defense compounds such as chromones succeeded in reducing the abundance of the fungus, leading to a weakening of the defense response of *A. sinensis* and a continued decrease in the rate of synthesis of chromones, which may be the main reason for the decrease in the content of chromones after a prolonged induction [39], or possibly due to the fact that the chromones are formed through a higher degree of hydroxylation or methoxylation to form other types of chromones that may not be detected [33]. This finding may help to distinguish agarwood samples with different formation times, and further studies on more agarwood samples with different formation times are needed to verify these conjectures.

## 4. Materials and Methods

### 4.1. Plant Materials

**Figure 6:**
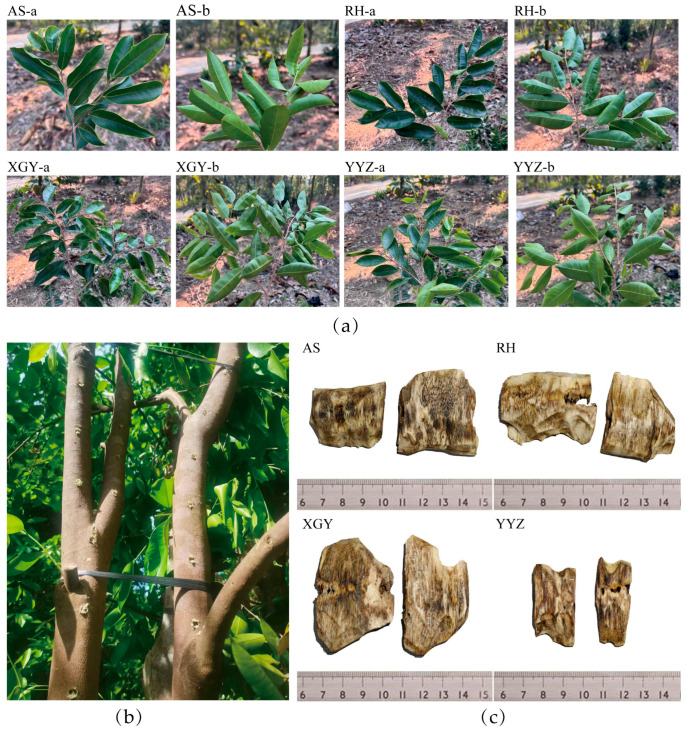
(**a**) Leaf morphology of the four “Qi-Nan” clones; (**b**) Artificial holes induced in agarwood after 3 years of cultivation of the AS clone. (**c**) The agarwood was induced for 12 months from four “Qi-Nan” clones. Note: AS-a and AS-b represent the front and back sides of the leaves of the AS clone, respectively, with similar naming conventions applied to the RH, XGY, and YYZ clones.

Four “Qi-Nan” agarwood clones from *A. sinensis*, named YYZ, AS, RH, and XGY, were selected by asexual propagation through grafting to produce superior germplasm more easily induced to produce agarwood [22]; Hu et al. demonstrated the distinct growth characteristics of the four clones [40]. They were planted in a plantation forest in the city of Dianbai in the Guangdong Province of China. The sampling site was located at 21°41′ N, 111°11′ E, at an altitude of 56 m above sea level. The agarwood trees were induced to produce agarwood by using an artificial fecundity technique. Three-year-old healthy Qi-Nan trees were chosen for agarwood induction, in which holes were drilled in the stem at 20 cm above the ground, with the distance between the holes being 8.0–10.0 cm, and the depth of the holes being two-thirds of the diameter of the trees (Figure 6), and the formation of agarwood resin was promoted by the artificial holing [41]. For each clone, five trees were sampled randomly at various locations to ensure representativeness for each duplication, three duplications were sampled for each induction time and clone. Previous studies informed the selection of 6, 12, and 24 months as representative time points for capturing the dynamic chemical composition changes during agarwood formation [19,20]. After 6, 12, and 24 months of induction, resin-rich wood was carefully harvested from the interior of the cut branches as specimens.

### 4.2. Methods

#### 4.2.1. Ethanol Extract

The sample was weighed 0.2 g (to 0.001 g precision) of crushed agarwood, placed in a stoppered centrifuge tube, and extracted by ultrasonic extraction using chromatographic grade ethanol (10 mL of 95% ethanol). Sonicate the mixture in a water bath at room temperature for 1 h using an ultrasonic extractor (P300H, Elma Schmidbauer GmbH, Singen, Germany) operating at a frequency of 37 kHz. After sonication, allow the mixture to cool, and then collect the supernatant and pass it through a 0.45 μm filter membrane to obtain the ethanol extracts for subsequent analysis, with three replicates of the samples [42].

#### 4.2.2. Determination of Chemical Composition

The samples were analyzed by gas chromatography-mass spectrometry (GC-MS) which was carried out using GC-Q-TOF-MS (8890–7250, Agilent Technologies, Santa Clara, CA, USA) equipped with a HP-5MS capillary column (30 m × 0.25 mm × 0.25 µm, Agilent Technologies). The initial oven temperature was set at 120 °C and then raised to 240 °C at a rate of 2 °C and kept at that temperature for 15 min.

Helium was the selected carrier gas used (flow rate = 1.0 mL·min^−1^). Samples were injected with a split ratio 10:1, injection volume was 1 µL. The electron impact ionization (EI) was 70 eV and the mass spectra were analyzed in the scan mode over the range of 33–550 amu. Samples were analyzed in triplicates. Compound identification was based on the fragmentation pattern of each compound that was matched with NIST-20 library installed on the instrument software. Compounds with mass spectra showing similarity index greater than 80% were taken in consideration. As well as the retention index (Kovat index) of each compound calculated after injection of a standard alkane series (C7-C40) under the same method and condition. Identification of the constituents was based on the comparison of their retention indices with those in the NIST Chemistry Webbook online library and previously reported RI of these compound in literature and by standard compounds. The standardized substances of 2-(2-phenylethyl)chromone, 6-hydroxy-2-(2-phenylethyl)chromone, and 2-[2-(4′-methoxybenzene) ethyl] chromone were obtained from Chengdu Push Bio-technology, Chengdu, China.

#### 4.2.3. Data Processing

All data are mean ± SD of three determinations. The data were normalized using log2 transformation and standardized with a z-score. We performed unsupervised analyses, including PCA and KMCA, as well as supervised analysis using OPLS-DA, to evaluate the metabolic profiling across groups. To validate the robustness of our models, permutation testing was conducted 200 times. Additionally, metabolites were visualized using volcano plots. All analyses were carried out using the R packages “prcomp”, “ropls”, “kmeans” and “ggplot2” in R version 3.5.1. Differential metabolites were screened by analyzing the VIP, FC, and *p*-value. One-way ANOVA and Two-way ANOVA (*p* < 0.05) were used to screen the significant variables with SPSS version 22.0.

## 5. Conclusions

We characterized the chemical composition of four “Qi-Nan” clones for the first time. The results revealed that the chemical properties of “Qi-Nan” agarwood were significantly influenced by the different clonal lines and induction times. The contents of PECs, SESs, and low-molecular-weight aromatic compounds varied considerably depending on the induction time and the specific “Qi-Nan” clone. The main chemical signatures of the four “Qi-Nan” agarwood clones were PECs, with exceptionally high contents of (46) and (52). Among the various types, the PECs in “Qi-Nan” agarwood were predominantly of the FTPECs type. Additionally, Eudesmanes and Eremophilanes were identified as the two main types of SESs present in the agarwood of the four “Qi-Nan” clones. Both the clonal lines of “Qi-Nan” agarwood and the induction time significantly influence the chemical composition of the agarwood, which in turn affects its quality and odor characteristics. After 24 months of induction, the RH, YYZ, and AS clones produced higher-quality agarwood compared to that obtained after 6 and 12 months of induction. In contrast, the XGY clone required a shorter induction period, achieving high-quality agarwood after just 12 months of induction. Different clonal lines and induction times exhibited distinct chemical properties and qualities of agarwood. The results of the study provide a valuable reference for the differentiation, harvesting application, and cultivation promotion of “Qi-Nan” agarwood in the future. It is also worth noting that the unique chemical characteristics of “Qi-Nan” agarwood may be related to the genetic information of the original plant germplasm or endophytic fungi, but the biosynthetic mechanisms of the above chemical properties need to be elucidated by further studies.

## Figures and Tables

**Table 1:** Chemical constituents detected in 3 induction time of “Qi-Nan” agarwood by GC-MS.

No.	Compound	Formula	RI ^a^	RI ^b^	Relative Content (%)											
					6 M				12 M				24 M			
					AS1	RH1	XGY1	YYZ1	AS2	RH2	XGY2	YYZ2	AS3	RH3	XGY3	YYZ3
**Agarofurans Sesquiterpenoids (2)**																
1	baimuxinol	C_15_H_26_O_2_	1767	1767	1.28	0.32	1.01	0.44	0.65	0.21	0.85	0.55	0.67	0.90	0.76	1.08
2	isobaimuxinol	C_15_H_24_O_2_	1846	1846	0.12	0.21	0.21	0.21	0.18	0.16	0.47	0.47	0.40	0.37	0.42	0.47
**Eremophilane Sesquiterpenoids (8)**																
3	eremophila-9,11(13)-dien-12-ol	C_15_H_24_O	1543	1540	0.29	0.89	0.17	0.70	2.07	0.52	0.94	0.55	1.01	0.43	0.88	0.59
4	valerianol	C_15_H_26_O	1676	1672	tr	0.10	0.08	0.12	0.23	tr	0.34	0.29	0.34	0.38	0.20	0.25
5	dihydrokaranone	C_15_H_22_O	1806	1813	0.55	0.31	0.49	0.36	0.47	0.37	1.30	0.75	0.87	1.18	1.21	1.36
6	hydroxyeremophilone	C_15_H_22_O_2_	1866	1865	0.39	0.61	0.33	0.44	0.74	0.47	0.58	0.46	0.59	0.58	0.57	0.54
7	valenca-1(10),8-dien-11-ol	C_15_H_20_O_2_	1878	1882	0.69	0.81	0.77	0.78	0.98	0.56	1.28	0.90	1.54	1.39	1.52	1.60
8	7α-*H*-9(10)-ene-11,12-epoxy-8-oxoeremophilane	C_15_H_22_O_2_	1911	1915	1.05	1.70	1.51	1.73	1.87	1.49	2.15	1.84	2.38	1.93	2.13	2.34
9	11-hydroxy-valenc-1(l0)-en-2-one	C_15_H_24_O_2_	1934	1978	0.32	0.51	0.71	0.49	0.57	0.57	1.11	0.60	0.98	0.86	0.83	0.87
10	isopetasol	C_15_H_22_O_2_	2026	2027	0.27	0.48	0.33	0.69	0.37	0.50	0.41	0.50	0.61	0.46	0.41	0.45
**Agarospirane Sesquiterpenoids (2)**																
11	agarospirol	C_15_H_24_O	1648	1646	0.17	0.13	0.15	0.17	0.22	0.16	0.23	0.21	0.25	0.17	0.24	0.19
12	baimuxinal	C_15_H_24_O_2_	1835	1830	0.10	0.33	tr	0.19	0.26	0.20	0.28	0.23	0.34	0.22	0.27	0.32
**Eudesmane Sesquiterpenoids (8)**																
13	eudesma-3,11(13)-dien-12-al	C_15_H_22_O	1703	1695	1.05	0.87	0.40	0.44	1.27	0.73	0.46	1.47	0.38	0.43	0.20	0.48
14	selina-4,11-dien-14-al	C_15_H_22_O	1793	1766	1.30	0.45	2.21	0.56	0.78	0.30	1.71	0.69	1.38	1.71	1.72	1.82
15	eudesma-5,11(13)-dien-8,12-olide	C_15_H_20_O_2_	1894	1890	0.59	0.72	0.60	0.79	1.02	0.67	0.33	0.54	0.26	0.35	0.29	0.39
16	6-(1-hydroxymethylvinyl)-4,8a-dimethyl-3,5,6,7,8,8a-hexahydro-1*H*-naphthalen-2-one	C_15_H_22_O_2_	1901	1909	0.46	0.31	0.57	0.33	0.50	0.19	0.12	0.11	0.15	0.32	0.18	tr
17	2(1*H*)-naphthalenone, 4a,5,6,7,8,8a-hexahydro-6-[1-(hydroxymethyl)ethenyl]-4,8a-dimethyl-,[4ar-(4aα,6α,8aβ)]-	C15H_22_O_2_	1923	1921	0.57	0.98	0.49	1.13	0.77	0.91	0.26	0.85	0.56	0.56	0.29	0.31
18	9-hydroxy-selina-4,11-dien-14-al	C_15_H_22_O_2_	2088	-	tr	0.16	0.46	0.15	tr	0.19	0.23	0.19	0.20	0.24	0.15	0.13
19	9-hydroxy-selina-3,11-dien-14-al	C_15_H_22_O_2_	2108	2097	tr	0.18	0.12	0.15	0.31	0.15	0.18	0.26	0.58	0.24	0.34	0.46
20	8,12-dihydroxy-selina-4,11-dien-14-al	C_15_H_22_O_3_	2249	2218	0.22	0.25	0.24	0.32	-	-	-	-	-	-	-	-
**Guaiane Sesquiterpenoids (6)**																
21	guaiol	C_15_H_26_O	1662	1614	0.09	0.26	0.09	0.18	0.90	0.14	0.10	0.35	0.56	0.27	0.37	0.32
22	guaia-1(10),11-dien-15-ol	C_15_H_24_O	1762	1770	0.48	0.19	tr	0.21	0.24	0.18	0.33	0.19	0.27	0.28	0.31	0.29
23	qinanol C	C_15_H_26_O_3_	1919	-	0.71	0.79	0.54	0.74	0.77	0.90	0.46	0.87	0.50	0.52	0.46	0.34
24	(3a*R*,4a*S*,5*R*,9a*S*)-5,8-dimethyl-3-methylene-3a,4,4a,5,6,7,9,9a octahydroazuleno [6,5-*b*]furan-2(3*H*)-one	C_15_H_20_O_2_	1929	1967	0.77	1.94	1.21	2.14	2.17	2.19	2.71	2.34	2.67	1.70	2.05	2.64
25	qinanol D	C_15_H_26_O_3_	2009	-	tr	0.32	0.27	0.36	0.22	0.42	0.21	0.29	0.19	0.17	0.25	0.22
26	guaia-1(10),11-diene-15,2-olide	C_15_H_20_O_2_	2168		0.94	0.81	0.59	0.85	1.10	0.91	0.96	0.93	0.79	0.72	0.61	0.88
**Other Sesquiterpenoids (5)**																
27	α-Santalol	C_15_H_24_O	1685	1681	1.55	0.46	0.82	0.40	1.39	0.17	1.68	1.04	1.70	1.44	1.89	1.68
28	β-Santalol	C_15_H_22_O	1711	1720	1.42	0.92	0.56	0.78	1.27	0.67	3.00	1.15	1.50	2.33	2.53	2.64
29	valerenol	C_15_H_24_O	1731	1736	0.55	0.71	0.55	1.32	1.47	0.64	1.22	1.15	0.97	0.95	1.30	1.13
30	dehydrosaussurea lactone	C_15_H_20_O_2_	1840	1838	0.47	0.62	0.69	0.76	0.54	0.66	0.93	0.79	0.88	0.51	0.91	1.07
31	ageratriol	C_15_H_24_O_3_	2092	2090	0.28	0.19	0.47	0.22	0.07	0.26	0.02	0.21	0.03	0.27	0.03	0.07
**Acids (1)**																
32	stearic acid	C_18_H_36_O_2_	2118	-	tr	0.11	tr	0.10	tr	0.14	tr	0.19	tr	tr	tr	tr
**Phenols (1)**																
33	4-pentylphenol	C_11_H_16_O	1515	1464	0.30	tr	0.15	tr	0.12	0.07	0.29	0.10	0.19	0.25	0.22	0.24
**Alcohols (1)**																
34	trans-sinapyl alcohol	C_11_H_14_O_4_	1953	-	tr	tr	tr	0.43	0.14	tr	tr	0.16	tr	tr	0.11	tr
**Esters(9)**																
35	α-kessyl acetate	C_17_H_28_O_3_	1787	1804	2.18	1.21	1.10	1.52	1.03	1.06	2.20	1.59	2.17	2.15	2.21	2.35
36	benzyl benzoate	C_14_H_12_O_2_	1831	-	2.07	0.60	1.06	0.65	0.80	0.40	1.74	1.07	1.75	1.35	1.51	1.40
37	2,3-dihydroxypropyl (9*Z*,12*Z*,15*Z*)-9,12,15-octadecatrienoate	C_21_H_36_O_4_	1884	-	tr	0.13	0.20	0.17	0.19	0.15	0.12	0.17	0.14	0.14	0.13	0.16
38	ethyl 2-oxo-4-phenylbutyrate	C_12_H_14_O_3_	1970	-	tr	0.17	tr	0.13	0.13	0.12	0.12	0.37	0.16	0.20	0.18	0.35
39	docosahexaenoic acid methyl	C_23_H_34_O_2_	1985	-	0.18	0.66	0.58	0.68	0.70	0.42	0.65	0.41	0.26	0.47	0.39	0.55
40	2-arachidonoylglycerol	C_23_H_38_O_4_	2053	-	0.04	0.08	0.09	0.11	0.06	0.08	0.11	0.14	0.01	0.01	tr	0.08
41	uvidin C, diacetate	C_19_H_30_O_5_	2062	-	tr	tr	0.15	0.16	0.05	0.11	0.10	0.15	tr	tr	0.10	0.10
42	benzenepropanoic acid, 2-phenylethyl ester	C_17_H_18_O_2_	2133	-	0.11	0.31	0.14	0.26	1.27	0.27	tr	1.48	0.11	0.12	tr	0.11
43	octadecanoic acid, ethyl ester	C_20_H_40_O_2_	2151	2195	0.24	0.23	0.14	0.26	0.43	0.30	tr	0.48	tr	0.21	0.10	tr
**Triterpenes(1)**																
44	Squalene	C_30_H_50_	2764	-	0.06	0.24	0.15	0.33	0.64	0.17	0.74	0.61	0.78	0.80	0.91	0.93
**Chromones(19)**																
45	2-methylchromone	C_10_H_8_O_2_	1488	-	1.94	0.69	1.06	0.65	0.58	0.58	0.77	0.48	0.73	0.66	0.90	0.75
46	2-(2-phenylethyl)chromone	C_17_H_14_O_2_	2269	2346	19.44	20.37	17.30	16.93	23.33	20.15	21.09	26.00	22.35	21.22	22.91	21.57
47	2-hydroxy-2-(2-phenylethyl)chromone	C_17_H_14_O_3_	2447	-	tr	0.25	tr	0.44	0.16	0.22	tr	0.74	tr	tr	tr	tr
48	6,8-dihydroxy-2-(2-phenylethyl)chromone	C_17_H_16_O_2_	2453	2458	0.62	0.34	0.23	1.07	0.67	0.58	tr	1.39	0.14	0.11	0.10	0.16
49	2-[2-(3-methoxy) phenylethyl]chromone	C_18_H_16_O_3_	2476	-	1.87	0.63	0.39	1.45	1.81	0.79	0.17	1.53	0.20	0.23	0.20	0.64
50	2-(2′-methoxyphenethyl)chromone	C_18_H_16_O_3_	2501	-	3.42	2.09	2.10	2.81	3.11	2.28	1.67	2.84	3.52	1.75	1.13	2.45
51	6-methoxy-2-(2-phenylethyl)chromone	C_18_H_16_O_3_	2527	2527	-	-	-	0.82	0.83	-	-	0.82	-	-	-	0.27
52	2-[2-(4-methoxyphenyl)ethyl]chromone	C_18_H_16_O_3_	2549	2545	16.38	19.26	20.80	19.38	17.74	20.48	20.51	19.07	19.86	22.98	22.23	20.81
53	agarotetrol	C_17_H_14_O_3_	2566	-	0.38	0.20	0.45	0.50	0.74	0.83	0.38	0.87	0.94	0.31	0.32	0.73
54	6,7-dimethoxy-2-[2-(4′-methoxyphenyl)ethyl]chromone	C_20_H_20_O_5_	2595	-	0.65	0.15	0.16	0.34	0.17	0.19	0.35	0.23	0.42	0.39	0.36	0.21
55	6-hydroxy-2-(2-phenylethyl)chromone	C_17_H_14_O_3_	2613	-	2.72	1.10	1.55	1.77	0.84	1.20	1.70	1.09	1.43	1.74	1.44	1.43
56	6-methoxy-2-[2-(4-methoxyphenyl)ethyl]chromone	C_19_H_18_O_4_	2628	2641	1.75	1.27	0.67	1.64	1.62	1.55	1.22	1.45	1.39	1.28	0.84	1.26
57	2-[2-(3-methoxy-4-hydroxyphenyl)ethyl]chromone	C_19_H_18_O_4_	2687	-	6.10	9.16	10.90	7.22	4.34	7.07	4.26	2.90	3.88	4.33	4.02	3.94
58	6-methoxy-7-hydroxy-2-[2-(4-methoxy)phenylethyl]-chromone	C_19_H_18_O_4_	2704	-	0.90	2.10	1.87	1.96	1.61	2.09	1.24	1.68	1.40	1.57	1.51	1.28
59	2-[2-(3-hydroxy-4-methoxyphenyl)ethyl]chromone	C_19_H_18_O_4_	2737	-	17.53	19.90	20.77	17.63	12.65	21.79	14.18	10.14	12.29	14.19	13.49	11.41
60	6-methoxy-2-[2-(3-methoxyphenyl)ethyl]chromone	C_19_H_18_O_4_	2785	-	tr	0.50	tr	0.86	0.41	0.53	0.42	0.43	0.47	0.57	0.50	0.67
61	6,7-dimethoxy-2-phenethylchromone	C_19_H_18_O_4_	2796	2822	2.82	0.22	0.42	0.22	0.13	0.42	0.25	0.36	1.01	0.18	0.10	0.54
62	6-hydroxy-2-[2-(4-methoxyphenyl)ethyl]chromone	C_19_H_18_O_4_	2808	-	0.75	0.11	0.55	0.12	tr	0.15	0.43	tr	0.32	0.56	0.49	0.37
63	6-hydroxy-2-[2-(3-hydroxy-4- methoxyphenyl)ethyl]chromone	C_19_H_20_O_4_	2908	-	0.17	0.18	0.15	1.15	0.13	0.18	0.11	0.23	0.13	0.15	0.14	0.13
the relative content of agarofurans sesquiterpenoids					1.39	0.53	1.23	0.65	0.83	0.37	1.32	1.02	1.08	1.27	1.19	1.54
the relative content of eremophilane sesquiterpenoids					3.56	5.41	4.39	5.32	7.29	4.48	8.12	5.89	8.32	7.22	7.75	8.00
the relative content of eudesmane sesquiterpenoids					4.19	3.93	5.07	3.88	4.65	3.14	3.28	4.12	3.52	3.84	3.17	3.59
the relative content of agarospirane sesquiterpenoids					0.27	0.46	0.15	0.36	0.48	0.36	0.51	0.44	0.59	0.39	0.51	0.51
the relative content of guaiane sesquiterpenoids					2.99	4.32	2.70	4.47	5.42	4.75	4.76	4.96	4.98	3.66	4.05	4.68
the relative content of acids					tr	0.11	tr	0.10	tr	0.14	tr	0.19	tr	tr	tr	tr
the relative content of phenols					0.30	tr	0.15	tr	0.12	0.07	0.29	0.10	0.19	0.25	0.22	0.24
the relative content of alcohols					tr	tr	tr	0.43	0.14	tr	tr	0.16	tr	tr	0.11	tr
the relative content of esters					4.82	3.38	3.46	3.94	4.65	2.89	5.04	5.87	4.60	4.65	4.62	5.08
the relative content of triterpenes					0.06	0.24	0.15	0.33	0.64	0.17	0.74	0.61	0.78	0.80	0.91	0.93
the relative content of chromones					77.43	78.54	79.37	76.95	70.86	81.09	68.74	72.22	70.51	72.23	70.69	68.61
Total					95.03	96.91	96.67	96.43	95.08	97.45	92.79	95.60	94.56	94.31	93.22	93.18

Note: RI ^a^ = Calculated Retention Index (HP-5MS column); RI ^b^ = Retention index of compounds retrieved from NIST Library; tr = Trace (Relative percent less than 0.01%); - = No detected; All compounds were identified by MS and RI in accordance with experimental protocols. AS1, AS2, and AS3 represent the AS clone treated for 6, 12, and 24 months, respectively, with similar naming rules applied to RH, XGY, and YYZ clone.

**Table 2:** Analysis of the main effect of PECs in “Qi-Nan” agarwood.

Source of Variance	SS	df	MS	*F* Value	*p* Value	Significance
Induction times	333.52	2	166.76	219.72	<0.000	***
Clonal line	120.29	3	40.10	52.83	<0.000	***
Induction times × Clonal line	142.24	6	23.71	31.24	<0.000	***
Errors	18.22	24	0.76	-		
Total variance	189,766.78	36				

Note: SS = Sum of Squares, df = degree of freedom, MS = Mean Square, *F* value = Variation between/within sample means, Asterisks (***) indicate significant differences at the *p* < 0.001 level.

## Data Availability

The data presented in this study are available in the text.
